# Job Demands, Stress Outcomes, and the Moderating Role of Resources Among Nursing Faculty in Saudi Arabia: A Cross-Sectional Study

**DOI:** 10.3390/healthcare14121629

**Published:** 2026-06-09

**Authors:** Norah M. Alyahya, Abdulaziz M. Alodhailah, Alya Alghamdi, Faihan F. Alshaibany, Majed M. Aljabri, Bandar S. Alharbi, Bader M. Almutairy, Safiya Salem Bakarman, Waleed M. Alshehri

**Affiliations:** 1 Community and Psychiatric Mental Health Nursing Department, College of Nursing, King Saud University, Riyadh 11451, Saudi Arabia; 2 Department of Medical-Surgical Nursing, College of Nursing, King Saud University, Riyadh 11451, Saudi Arabia; 3 Department of Nursing Administration and Education, College of Nursing, King Saud University, Riyadh 11451, Saudi Arabia

**Keywords:** burnout, professional [MeSH], faculty, nursing [MeSH], job satisfaction [MeSH], occupational stress [MeSH], Saudi Arabia [MeSH], surveys and questionnaires [MeSH], workplace [MeSH], Job Demands–Resources model

## Abstract

Background: Nursing faculty shortages, burnout, and high turnover represent an escalating workforce crisis in Saudi governmental colleges of nursing. The Job Demands–Resources (JD-R) model offers a theoretically grounded framework for examining how occupational demands are associated with reduced well-being and how resources moderate these effects. Objective: This study aimed to examine the direct associations between job demands and stress outcomes and the moderating roles of job and personal resources among nursing faculty in Saudi Arabia, accounting for gender and nationality as structural covariates. Methods: A quantitative cross-sectional survey was conducted with 268 nursing faculty members from five governmental colleges using a voluntary survey of all eligible faculty (response rate: 51.1%). Theory-driven hierarchical regression analyses examined direct and moderating effects within the health-impairment pathway of the JD-R model. Results: Job demands significantly predicted all three burnout dimensions, reduced mental well-being, and job dissatisfaction. Trait emotional intelligence moderated the demand–exhaustion (delta-R^2^ = 0.031, *p* = 0.006) and demand–job satisfaction (delta-R^2^ = 0.028, *p* = 0.009) relationships. Job resources moderated the demand–mental well-being (delta-R^2^ = 0.024, *p* = 0.018) and demand–professional efficacy links (delta-R^2^ = 0.021, *p* = 0.029). Conclusions: Job demands are the primary predictor of burnout and occupational stress. Gender and nationality were associated with systematic differences in stress outcomes, suggesting that interventions should be culturally responsive and account for structural inequities.

## 1. Introduction

The global shortage of nursing faculty is a well-documented and escalating crisis. Estimates indicate that shortfalls in academic nursing staff constitute a primary bottleneck in efforts to expand nursing graduate pipelines, a concern acutely felt in countries undergoing rapid demographic and healthcare transformation [1]. Saudi Arabia exemplifies this challenge: the Kingdom’s Vision 2030 agenda commits to expanding nursing education capacity, yet governmental nursing colleges struggle to recruit and retain qualified academic staff [2]. With only 29.1% of the clinical nursing workforce comprising Saudi nationals and nursing faculty positions increasingly dependent on fixed-term expatriate contracts, the academic pipeline for nursing in Saudi Arabia remains structurally fragile [3,4].

Burnout and its sequelae, including reduced job satisfaction, deteriorating mental well-being, and ultimately voluntary turnover, represent the primary mechanisms through which workforce capacity may be reduced [5]. The consequences extend beyond individual health: burnout among academic nursing faculty compromises teaching quality, research productivity, and the long-term sustainability of nursing education systems [6]. Yet the antecedents of faculty burnout in a Saudi context have not been systematically examined. Much of the existing literature on nursing faculty well-being derives from North American and Western European settings, where employment conditions, gender roles, and organisational cultures differ substantially from those in Gulf Cooperation Council (GCC) states [7].

The Job Demands–Resources (JD-R) model [8] offers a theoretically versatile and empirically robust framework for analysing the dual processes through which occupational characteristics affect employee outcomes. The model posits that job demands, encompassing the physical, psychological, social, or organisational aspects of work that require sustained effort and are associated with physiological and psychological costs, initiate a health-impairment process leading to burnout. Conversely, job and personal resources fuel a motivational process that sustains engagement and buffers demands. The model’s dual-process architecture has been validated extensively across health professions internationally [9], yet its application to nursing academia in the Arab world remains sparse. Psychosocial hazards in the workplace, as recognised by the World Health Organization, encompass precisely the types of demands that characterise academic nursing environments, including high workload, role ambiguity, and limited control [2].

Saudi nursing academia introduces contextual variables largely absent from Western studies. Gender segregation in educational settings means that female faculty face compounded role demands at the intersection of professional expectations and domestic responsibilities shaped by cultural norms. The guardianship system, though substantially modified under Vision 2030 reforms, continues to influence women’s career experiences [10]. Furthermore, the coexistence of Saudi national and expatriate faculty within the same colleges creates a differentiated occupational environment in which career trajectories, contractual stability, and organisational commitment diverge markedly [11]. Studies have documented elevated burnout rates among nursing staff working in Saudi healthcare contexts, suggesting that both clinical and academic nursing environments carry substantial occupational risk [12].

These structural features suggest that demand–stress pathways and resource-buffering effects may operate differently in this context than models derived from homogeneous Western populations would predict. Despite its theoretical relevance, only a limited body of research has tested the JD-R model specifically with nursing faculty [13], and to our knowledge, none has done so in Saudi Arabia or comparable GCC contexts. The present study addresses this gap by pursuing three specific objectives: (1) to examine the direct associations between job demands and burnout, mental well-being, and job satisfaction; (2) to test the moderating effects of job and personal resources on these demand–outcome relationships; and (3) to determine whether gender and nationality function as significant structural covariates of stress outcomes after controlling for demand levels. Addressing these objectives extends JD-R evidence to an underrepresented context and generates findings relevant to nursing workforce policy in Saudi Arabia and comparable GCC settings.

## 2. Theoretical Framework

The JD-R model [9] conceptualises the work environment through two broad categories of occupational characteristics. Job demands encompass workload, emotional demands, role conflict, and role ambiguity. Job resources include supervisor support, colleague relationships, autonomy, and performance feedback. Personal resources, defined as positive self-evaluations linked to resilience and the capacity to control and influence the environment, include constructs such as self-efficacy, optimism, and emotional intelligence [8]. Trait emotional intelligence, in particular, represents a constellation of emotion-related self-perceptions and dispositions that shape how individuals appraise and respond to occupational stressors [14].

Two distinct psychological mechanisms are proposed within the model. The health-impairment pathway holds that chronic job demands deplete cognitive and emotional reserves, culminating in exhaustion, cynicism, and ultimately burnout. The motivational pathway contends that job and personal resources foster intrinsic motivation, goal attainment, and work engagement. Crucially, the model allows for interactive effects: resources are hypothesised to buffer the deleterious impact of demands, an interaction effect that has been demonstrated across multiple health profession contexts [6]. Meta-analytic evidence confirms that this buffering effect operates across diverse occupational settings, including those characterised by high safety and care demands [5].

The present study tests the health-impairment (negative) pathway only. The motivational pathway is described for theoretical completeness but is not empirically tested herein, which constitutes a stated limitation and avenue for future research.

Job Demands (Workload, Role Conflict, Emotional Demands) → Health-Impairment Process [Moderated by Resources] → Stress Outcomes (Burnout, Reduced Well-being, Low Job Satisfaction). Job Resources (Support, Autonomy, Feedback) and Personal Resources (Emotional Intelligence, Self-efficacy) → Motivational Process → Engagement and Commitment/Intention to Remain.

## 3. Methods

### 3.1. Study Design and Setting

A cross-sectional survey design was employed to examine associations between job demands, resources, and stress outcomes at a defined point in time, acknowledging that this design precludes causal inference [15]. Data were collected from five governmental colleges of nursing in Saudi Arabia between 2019 and 2020, selected purposively to represent diverse geographic regions and variability in institutional size. The study was reported in accordance with the Strengthening the Reporting of Observational Studies in Epidemiology (STROBE) checklist for cross-sectional studies.

### 3.2. Participants and Sampling

A total-population sampling strategy was employed, inviting all eligible nursing faculty at the five sites to participate. Eligibility required current employment (full-time or part-time); faculty on unpaid leave or with primary employment elsewhere were excluded. Of 524 eligible faculty identified, 268 returned completed questionnaires (response rate: 51.1%), constituting a partial census rather than a complete enumeration; non-response bias cannot be ruled out, and a systematic respondent comparison was not feasible. Site-level response rates ranged from 44% to 61%. Questionnaire packages were distributed via department heads at each site and collected in sealed envelopes to maintain confidentiality. Twelve cases with incomplete scale data were excluded prior to analysis using listwise deletion. A post-hoc power analysis (G*Power version 3.1.9.7; multiple regression, R^2^ = 0.254, 12 predictors, N = 268, alpha = 0.05) confirmed achieved power of 0.99 for the primary model; for moderation models (delta-R^2^ approximately 0.02–0.03), power ranged from 0.72 to 0.81.

### 3.3. Measures

Job demands and job resources were assessed using the Questionnaire on the Experience and Evaluation of Work (QEEW-2) [16,17], a widely validated Dutch-origin instrument with established psychometric properties across cross-cultural applications. In the present study, a Saudi-Arabic version was developed through a systematic adaptation process. The original Dutch instrument was translated into Arabic by two bilingual nursing academics, independently back-translated into English by a third bilingual translator, and discrepancies were resolved through expert consensus. Cognitive interviews were conducted with a pilot sample of eight nursing faculty members (not included in the main study) to assess item clarity, cultural appropriateness, and response format comprehension. Pilot testing on a separate convenience sample (*n* = 25) confirmed satisfactory internal consistency for all subscales prior to main data collection (alpha range 0.78–0.87). Formal psychometric equivalence testing was not conducted; this represents a stated limitation. Subscales measuring demands included quantitative workload, emotional demands, role conflict, and role ambiguity. Subscales measuring resources included colleague support, supervisor support, autonomy, performance feedback, and opportunities for development.

Burnout was measured using the Maslach Burnout Inventory General Survey (MBI-GS) [18], which yields three subscale scores: emotional exhaustion (MBI-EX), cynicism (MBI-CY), and professional efficacy (MBI-PE). Higher scores on exhaustion and cynicism indicate greater burnout; professional efficacy is reverse-scored for analysis in the negative arm. The MBI-GS was selected over alternative burnout instruments [19] because of its widespread use and established psychometric properties across academic and health professional populations.

Mental well-being was measured using the Warwick–Edinburgh Mental Well-being Scale (WEMWBS) [20], a 14-item scale assessing positive aspects of mental health with strong psychometric properties across adult populations, including non-UK samples [20]. Job satisfaction was assessed with the Job Descriptive Index (JDI) [21], covering five facets: work on present job, present pay, opportunities for promotion, supervision, and coworkers. Personal resources were assessed using the Trait Emotional Intelligence Questionnaire-Short Form (TEIQue-SF) [22], a 30-item measure of trait emotional intelligence with satisfactory reliability across diverse cultural samples [22].

### 3.4. Data Analysis

Data were analysed using IBM SPSS Statistics version 25. Scale reliability was assessed via Cronbach’s alpha. Normality was assessed using the Kolmogorov–Smirnov test. Square-root transformation was applied to MBI-EX prior to regression analysis; WEMWBS was analysed on its original scale after normality assessment did not indicate severe violation. The reference to log-10 transformation for ‘work engagement’ in a prior version was an error and has been removed; work engagement was not assessed in this study. Non-parametric tests (Mann–Whitney U; Kruskal–Wallis H) were used for group comparisons. Variance inflation factors (VIF; all below 3.0) confirmed the absence of multicollinearity across all regression models. Homoscedasticity was assessed via residual plot inspection; no systematic violations were observed.

Theory-driven hierarchical regression was employed to examine the health-impairment pathway of the JD-R model. Block entry was determined by theoretical logic: demographic and professional covariates (gender, nationality, age, academic experience) entered Block 1, and total job demands entered Block 2.

## 4. Results

### 4.1. Sample Characteristics

Of the 268 participants, 71.3% (*n* = 191) were female and 28.7% (*n* = 77) were male. Saudi nationals comprised 52.6% (*n* = 141) of the sample; non-Saudi expatriates comprised 47.4% (*n* = 127). The mean age was 38.6 years (SD = 8.4). The mean duration of experience in academia was 9.2 years (SD = 6.1). Faculty were distributed across all five sites, with site-level response rates ranging from 44% to 61%. Detailed demographic and professional characteristics are presented in Table 1.

### 4.2. Descriptive Statistics and Scale Reliability

Table 2 presents descriptive statistics and Cronbach’s alpha coefficients for all study measures. All scales demonstrated acceptable to excellent internal consistency (alpha ranging from 0.74 to 0.93). Mean burnout scores indicated moderate levels of emotional exhaustion (M = 2.81, SD = 1.22) and cynicism (M = 2.34, SD = 1.18), with professional efficacy scores suggesting moderate perceived competence (M = 4.12, SD = 0.98). Mental well-being scores (WEMWBS) were in the moderate range (M = 45.6, SD = 9.1). Job satisfaction (JDI total) indicated moderate overall satisfaction, with the lowest facet scores observed for promotion opportunities and pay.

### 4.3. Group Differences in Stress Outcomes

Gender differences were significant for emotional exhaustion (U = 5842, *p* < 0.001) and job satisfaction (U = 5221, *p* = 0.003), with female faculty reporting higher exhaustion and lower job satisfaction than male colleagues. No significant gender difference was found for cynicism (*p* = 0.07) or mental well-being (*p* = 0.12). Nationality differences were significant across all stress outcomes: Saudi nationals reported significantly higher emotional exhaustion (U = 6341, *p* < 0.001), higher cynicism (U = 6018, *p* = 0.002), lower mental well-being (U = 5934, *p* < 0.001), and lower job satisfaction (U = 5711, *p* = 0.001) than non-Saudi faculty. Older participants (aged 40 years or above) and those with longer academic experience (10 years or more) reported significantly lower burnout and higher mental well-being than their younger and less experienced counterparts (*p* < 0.05 for all comparisons). Group differences in stress outcomes are presented in Table 3.

### 4.4. Regression Analyses: Negative Arm of the JD-R Model

Consistent with H1, theory-driven hierarchical regression confirmed that job demands made a significant independent contribution to each stress outcome across all five models (predicting MBI-EX, MBI-CY, MBI-PE, WEMWBS, and JDI) after controlling for demographic covariates. Consistent with H2, female gender independently predicted greater emotional exhaustion (beta = 0.21, *p* = 0.001) and lower job satisfaction (beta = −0.18, *p* = 0.003); Saudi nationality predicted higher cynicism (beta = 0.17, *p* = 0.008) and lower mental well-being (beta = −0.19, *p* = 0.004). The full hierarchical regression model for emotional exhaustion is presented in Table 4. Hierarchical regression results for the remaining four dependent variables (MBI-CY, MBI-PE, WEMWBS, and JDI) are presented in which report all blocks, standardised coefficients, delta-R^2^ values, and interaction terms for each model.

### 4.5. Moderating Effects of Resources

The Johnson–Neyman procedure identifies the values of the moderator at which the slope of the predictor transitions from non-significant to significant. Consistent with H3, personal resources (trait EI) significantly moderated the demand–exhaustion relationship (delta-R^2^ = 0.031, *p* = 0.006), and consistent with H4, also moderated the demand–job satisfaction relationship (delta-R^2^ = 0.028, *p* = 0.009). Probing the significant interaction for emotional exhaustion revealed that the demand–exhaustion association was substantially attenuated at high levels of personal resources.

These moderation effects are small in magnitude (delta-R^2^ = 0.02–0.03), consistent with typical field–study interaction effect sizes in occupational health and are discussed further in Section 5. Consistent with H5 and H6, these findings collectively support the buffering hypothesis within the JD-R model for most, but not all, stress outcome pathways.

## 5. Discussion

### 5.1. Principal Findings

This study provides the first multi-site empirical test of the JD-R model’s negative arm within Saudi nursing academia, and the results broadly support the model’s predictions. Job demands were the strongest predictor of all three burnout dimensions, mental well-being, and job satisfaction, findings consistent with the model’s health-impairment hypothesis and with prior evidence from clinical nursing contexts [21,23,24]. The magnitude of demands’ predictive contribution (beta = 0.42 for emotional exhaustion) falls within the range reported in international nursing faculty studies [25], supporting the cross-cultural generalisability of this pathway. These findings also align with meta-analytic evidence demonstrating robust demand-to-burnout associations across health and social care professions [9,26].

### 5.2. Gender and Nationality as Structural Amplifiers

These structural conditions plausibly intensify job demands for women over and above the measured workload indicators, which may explain why gender remained a significant predictor after controlling for reported demands; domestic responsibilities and gender-role expectations were not directly measured and remain interpretative hypotheses. The psychological burden of managing such intersecting responsibilities is well recognised in occupational health literature.

The nationality difference, specifically Saudi nationals reporting worse stress outcomes than non-Saudi colleagues, warrants careful interpretation. Saudi nationals occupy a structurally different position within nursing colleges: they hold permanent positions with expectations of longer tenure, may carry heavier administrative and national development obligations, and face a professional nursing culture that, despite improvements, continues to struggle with social prestige and identity in the local context [27]. Non-Saudi faculty, while often on fixed-term contracts, bring established professional identities from international nursing systems and may be better insulated from the cultural tensions that characterise nursing’s evolving social standing in Saudi Arabia. Empowerment through clear role definition and participatory management structures may help to address these disparities [5].

### 5.3. Moderating Effects of Resources

The significant moderating effects of both personal and job resources support the model’s buffering hypothesis, though with important nuances. Trait emotional intelligence most strongly buffered the demand–exhaustion and demand–job satisfaction links, suggesting that the capacity to perceive, understand, and regulate emotions functions as a genuine psychological buffer under conditions of high occupational demand. This finding aligns with recent meta-analytic evidence linking trait EI to occupational stress resilience across health professions [28] and reinforces the rationale for emotionally oriented faculty development programmes. Earlier foundational work on trait EI similarly demonstrated its role in moderating affective responses to occupational stressors [29], lending theoretical support to the present findings.

The absence of significant moderation for the demand–cynicism link may reflect the more cognitively mediated nature of cynicism as a burnout dimension. Cynicism, as a form of psychological distancing from work, may respond less readily to emotional regulation capacities than does the affective dimension of exhaustion. This differential pattern is consistent with multi-dimensional conceptualisations of burnout [30] and suggests that interventions targeting cynicism may need to address cognitive appraisal processes rather than emotional regulation alone. Job resource buffering, demonstrated for mental well-being and professional efficacy, underscores the organisational responsibility to provide supportive supervisory relationships, meaningful feedback, and opportunities for professional development, all elements known to sustain nurse faculty engagement [18,31].

### 5.4. Practical Implications

In terms of practical implications at the individual level, these findings support investment in trait EI development as a component of faculty well-being programmes. At the department level, workload equity audits are warranted, with particular attention to female faculty who carry disproportionate occupational burden. At the institutional level, differentiated support for Saudi national faculty is indicated, including mentorship programmes, clearer career progression pathways, and recognition of disproportionate administrative obligations. Transferability to other GCC contexts is plausible but requires verification through replication. 

### 5.5. Strengths and Limitations

This study offers several methodological strengths: its multi-site design, use of validated instruments, and rigorous theory-driven moderation testing [30]. The sample of 268 provided adequate statistical power for the primary regression analyses. Limitations include: the cross-sectional design precluding causal inference; the 51.1% response rate and non-response bias risk; restriction to governmental colleges; operationalisation of personal resources through trait EI alone; total-score aggregation potentially obscuring dimension-specific effects; absence of formal site-level clustering; and the COVID-19 temporal overlap. Future research should employ longitudinal designs, include private college samples, test the full JD-R model using structural equation modelling, and conduct intervention trials for emotional intelligence programmes.

## 6. Conclusions

Female gender and Saudi nationality were associated with systematically worse stress outcomes, suggesting that universal workforce interventions are unlikely to be sufficient and that context-sensitive, equity-informed approaches are warranted. Targeted support, including trait EI development programmes, equitable workload allocation, and role clarity initiatives, may help protect faculty well-being. Resource provision, both job-based and personal, moderated these harmful pathways, suggesting it may be a viable focus for retention-oriented workforce policies.

These findings contribute empirical evidence from an under-researched context to the growing international literature on nursing faculty well-being, and affirm the utility of the JD-R model as an explanatory and policy-relevant framework beyond its Western origins. As Saudi Arabia continues to invest in nursing education under Vision 2030, ensuring that the academic nursing workforce is protected from burnout and supported through adequate resources is essential for the long-term sustainability of the nursing profession in the Kingdom.

## Figures and Tables

**Table 1:** Demographic and professional characteristics of participants (N = 268).

Characteristic	*n*	%
Gender		
Female	191	71.3
Male	77	28.7
Nationality		
Saudi national	141	52.6
Non-Saudi expatriate	127	47.4
Age category		
<40 years	152	56.7
≥40 years	116	43.3
Academic experience		
<10 years	148	55.2
≥10 years	120	44.8
Academic rank		
Lecturer/Instructor	98	36.6
Assistant Professor	89	33.2
Associate/Full Professor	81	30.2

Note. Age and academic experience are presented as categorical distributions per the original data collection format. Continuous means are reported in the text: age M = 38.6 years (SD = 8.4); academic experience M = 9.2 years (SD = 6.1). Categorical presentation was retained for comparative group analyses; reviewer feedback is acknowledged, and future reports will present continuous variables using M and SD as the primary summary statistic.

**Table 2:** Descriptive statistics and internal consistency for study measures (N = 268).

Scale/Subscale	M	SD	Range	Cronbach’s Alpha
QEEW-2 Total Job Demands	2.98	0.61	1–5	0.86
QEEW-2 Total Job Resources	3.21	0.57	1–5	0.88
MBI-EX (Emotional Exhaustion)	2.81	1.22	0–6	0.91
MBI-CY (Cynicism)	2.34	1.18	0–6	0.88
MBI-PE (Professional Efficacy)	4.12	0.98	0–6	0.82
WEMWBS (Mental Well-being)	45.6	9.1	14–70	0.89
JDI Total (Job Satisfaction)	127.4	28.6	0–210	0.87
TEIQue-SF (Trait EI)	4.71	0.84	1–7	0.85

**Table 3:** Group differences in stress outcomes by gender, nationality, age, and experience.

Variable	MBI-EX	MBI-CY	WEMWBS	JDI	*p*
Female (*n* = 191)	3.06 (1.18)	2.41 (1.19)	44.2 (9.4)	121.3 (29.1)	<0.001 *
Male (*n* = 77)	2.22 (1.19)	2.18 (1.14)	48.8 (8.2)	141.6 (24.8)	<0.001 *
Saudi national (*n* = 141)	3.14 (1.21)	2.58 (1.22)	42.8 (9.6)	118.2 (28.4)	<0.001 *
Non-Saudi (*n* = 127)	2.44 (1.16)	2.07 (1.10)	48.8 (8.1)	137.9 (27.2)	<0.001 *
Age < 40 (*n* = 152)	3.02 (1.24)	2.48 (1.21)	44.1 (9.3)	123.1 (28.8)	<0.05 *
Age ≥ 40 (*n* = 116)	2.53 (1.16)	2.14 (1.12)	47.7 (8.6)	133.2 (28.1)	<0.05 *

Note. Values are M (SD). MBI-EX = Maslach Burnout Inventory Emotional Exhaustion; MBI-CY = Cynicism; WEMWBS = Warwick–Edinburgh Mental Well-being Scale; JDI = Job Descriptive Index. * Significant at *p* < 0.05.

**Table 4:** Hierarchical stepwise regression: Dependent Variable—Emotional Exhaustion (MBI-EX, SQRT-transformed), N = 268.

Predictor	B	SE B	Beta	t	*p*
Block 1: Demographics					
Gender (female = 1)	0.18	0.06	0.21	3.00	0.003
Nationality (Saudi = 1)	0.11	0.05	0.15	2.20	0.029
Age (years)	−0.02	0.01	−0.12	−2.00	0.047
Block 2: Job Demands					
Total Job Demands (QEEW-2)	0.34	0.05	0.42	6.80	<0.001
Delta R^2^ (Block 2)			0.176		<0.001
Total R^2^			0.254		<0.001

## Data Availability

The datasets presented in this article are not readily available because the data are part of an ongoing study. Data sharing is restricted under the approved ethics protocol.
